# *Trypanosoma vivax* Infections: Pushing Ahead with Mouse Models for the Study of *Nagana*. II. Immunobiological Dysfunctions

**DOI:** 10.1371/journal.pntd.0000793

**Published:** 2010-08-10

**Authors:** Marie Christine Blom-Potar, Nathalie Chamond, Alain Cosson, Grégory Jouvion, Sabrina Droin-Bergère, Michel Huerre, Paola Minoprio

**Affiliations:** 1 Laboratoire d'Immunobiologie des Infections à Trypanosoma, Département d'Immunologie, Institut Pasteur, Paris, France; 2 Unité de Recherche et d'Expertise Histotechnologie et Pathologie, Institut Pasteur, Paris, France; New York University School of Medicine, United States of America

## Abstract

*Trypanosoma vivax* is the main species involved in trypanosomosis, but very little is known about the immunobiology of the infective process caused by this parasite. Recently we undertook to further characterize the main parasitological, haematological and pathological characteristics of mouse models of *T. vivax* infection and noted severe anemia and thrombocytopenia coincident with rising parasitemia. To gain more insight into the organism's immunobiology, we studied lymphocyte populations in central (bone marrow) and peripherical (spleen and blood) tissues following mouse infection with *T. vivax* and showed that the immune system apparatus is affected both quantitatively and qualitatively. More precisely, after an initial increase that primarily involves CD4^+^ T cells and macrophages, the number of splenic B cells decreases in a step-wise manner. Our results show that while infection triggers the activation and proliferation of Hematopoietic Stem Cells, Granulocyte-Monocyte, Common Myeloid and Megacaryocyte Erythrocyte progenitors decrease in number in the course of the infection. An in-depth analysis of B-cell progenitors also indicated that maturation of pro-B into pre-B precursors seems to be compromised. This interferes with the mature B cell dynamics and renewal in the periphery. Altogether, our results show that *T. vivax* induces profound immunological alterations in myeloid and lymphoid progenitors which may prevent adequate control of *T. vivax* trypanosomosis.

## Author Summary

*Trypanosoma vivax* is responsible for animal trypanosomosis, or *Nagana*, in cattle and small ruminants. Under experimental conditions, the outbred mouse model infected with a well studied West African *T. vivax* isolate reproduces the main characteristics of the infection and pathology observed in livestock. Anemia and non-specific (parasite-directed) polyclonal hypergammaglobulinemia are the most common disorders coincident with the rise in parasitemia. Our results presented here show that the decrease in peripheral B cell populations does not seem to be compensated by newly arriving B cells from the bone marrow. The infection nevertheless prompts intense production of stem cells that mature into myeloid and lymphoid precursors. In spite of this, B cell numbers are specifically reduced in the periphery as the infection progresses. Thus, negative feedback seems to be set in motion by the infection in the bone marrow, more precisely affecting the maturation of B precursors and consequently the output of mature B cells. The origin of these phenomena is unclear but this doubtless creates a homeostatic imbalance that contributes to the inefficient immune response against *T. vivax* infection.

## Introduction

African trypanosomes are extracellular parasites that cause sleeping sickness in humans and *Nagana* in animals. They include *T. brucei* species which infect both humans and ruminants, but also *T. congolense* and particularly *T. vivax* which are responsible for the vast majority of animal trypanosomosis in sub-Saharan Africa, South America and South Asia [1]–[3]. Due mainly to technical constraints such as a lack of reproducible *in vitro* culture conditions and relatively poor accessibility to natural hosts, our understanding of the biology and fate of *T. vivax* in its vertebrate hosts largely stems from the extrapolation of data obtained from the experimental murine infection with *T. brucei*, *T. congolense* and *T. ewansi*, but just a few studies using *T. vivax* infected mice [4]–[8]. Recently, in a move to gain further insight into the host - *T. vivax* interaction, we further developed reproducible and reliable *in vivo* models of *T. vivax* infection using three different mouse strains and the IL 1392 West African isolate (*see accompanying paper*). Briefly, our studies showed that all the mouse strain infected with bloodstream forms of *T. vivax* developed the characteristic anemia and systemic alterations that include acute necrosis of the liver and spleen which are the hallmarks of animal trypanosomosis [9]–[12].

Previous immunobiological studies of trypanosomosis focused mainly on the interaction between trypanosome surface coat antigens (Variant Surface Glycoproteins, VSGs) and host cells [13]–[15]. The triggering of polyclonal B cell activation by trypanosomes and the ensuing hypergammaglobulinemia mainly composed of antibodies (Ab) that do not recognize parasite antigens or VSGs are also typical of the infection [16]–[19]. The mechanisms underlying this process are still largely unknown. Moreover, the involvement of VSGs in protecting the parasites against host specific immunoresponses provided until recently one of the most exquisite models for the study of antigenic variation. It therefore followed, for many years, that our understanding of the interaction between African trypanosomes and the immune system was limited to this “parasite-driven” view where the host's immune response was restricted to the production of specific Abs against VSGs. Whereas anti-VSG Ab doubtless contribute to early control of the infection, resistance to late phases is not only dependent on specific (parasite-directed) immunoglobulins but also seems to rely on T-independent processes since athymic mice and also complement-deficient mice infected with *T. rhodesiense* are able to mount anti parasitic responses that are sufficient to increase mouse survival and healing after an infectious challenge [7], [20]. Interestingly, the severity of the disease correlates with the control exerted by *T. brucei-* and *T. congolense-* specific Abs over the frequency and duration of parasitemia waves but not the level of circulating parasites. This contrasts with *T. vivax* infections where the efficiency of the host's Ab response and the parasite-induced negative feedback of Abs raised against the parasite are responsible for regulating both the level and duration of parasitemia waves, thus determining disease severity [21].

In an attempt to throw light on the early events induced by *T. vivax* in mouse B cell compartments that may contribute to explaining later disturbances in the peripheral B cell pool, we studied the impact of parasite infection both on bone marrow and peripheral lymphoid tissues. Our results using an outbred strain showed that *T. vivax* mouse infection readily results in B cell differentiation accompanied by massive production of polyclonal immunoglobulins that are mostly nonspecific of parasite antigens. The infection profoundly disorganized the follicular structure of the spleen and similarly to *T. brucei* infection [6] appeared to destroy the B cell marginal zone, certainly contributing to the substantial fall in B cell population numbers both in the spleen and blood. Numbers of both marginal zone and follicular B cells decreased in the organ in the course of the infection concomitantly with a rise in plasmocytes. Bone marrow analysis showed a sustained and significant increase in the long–term, self-renewal of stem cells on infection. However, it is the fate of B cells, and more precisely of B-cell precursors, that seems to be particularly affected, and this impacts on B cell output. The long-lasting destruction of the spleen marginal zone by *T. brucei* was previously shown to constitute a barrier to the development of efficient B cell memory and thus to a vaccine-induced B cell response [6]. Our results presented here further indicate that *T. vivax* infection disturbs the development of B cells in bone marrow which could be an obstacle to immunotherapies against trypanosomosis and even impair unrelated vaccination strategies in trypanosome-exposed populations [18].

## Materials and Methods

### Mice, parasites and infection

*Trypanosoma vivax* isolate ILRAD 1392 was kindly provided by R. Brun (Swiss Tropical Institute, Basel, Switzerland). The phenotypic and molecular identification of this isolate has previously been described (see accompanying paper). Bloodstream forms of *T. vivax* were maintained by weekly passages in 7- to 8-week-old outbred RjOrl:Swiss mice (CD-1, Janvier, France) by intra-peritoneal (i.p.) injection of 10^3^ bloodstream forms. Outbred mice were chosen as the experimental model since they present significantly higher survival rates than BALB/c mice and, as described before, a detailed histopathological study of the organs committed by the infection showed similar pathognomonic signs of the disease to those observed in the infected livestock (see accompanying paper). Furthermore, parasitemia in outbred mice reached a plateau by day 10 of the infection and this persisted over time contrasting with the more tolerant C57BL/6 mice that showed recurrent waves of parasitemia. Thus, in our model, 7- to 10-week-old male outbred CD-1 mice were infected i.p. with 10^2^ bloodstream parasites. Parasitemia was determined every 2 to 3 days using a counting chamber and a light microscope as described (see accompanying paper). The experiments described here were performed every 3–5 days throughout the infection, but for clarity and without any loss of important information, only days 10 and 20 post infection are portrayed and considered in the present work since they correspond respectively to peak parasitemia in outbred mice and to the day that generally precedes death, respectively. All animal work was conducted in accordance with relevant national and international guidelines (see here below).

### Ethics statement

All mice were housed in our animal care facilities in compliance with European animal welfare regulations. The Institut Pasteur is member of the Committee #1 of the Comité Régional d′Ethique pour l′Expérimentation Animale (CREEA), Ile de France. The Animal housing conditions and protocols used in the present work were previously approved by the “Direction des Transports et de la Protection du Public, Sous-Direction de la Protection Sanitaire et de l'Environnement, Police Sanitaire des Animaux” under the number #B 75-15-28 accordingly to the Ethics Chart of animal experimentation which includes appropriate procedures to minimize pain and animal suffering. PM has permission to perform experiments on vertebrate animals #75-846 issued by the Department of Veterinary Services of Paris, DDSV and is responsible for all the experiments and protocols carried out personally or under her direction in the framework of laws and regulations relating to the protection of animals.

### Parasite extracts

Bloodstream forms of *T. vivax* were collected by cardiac punction then diluted in buffer A (polysome buffer) before being centrifuged for 5 min at 1000 rpm to separate the parasites from the red blood cells. The upper phase was collected and the pellet washed twice. Supernatants were recovered and pooled. Parasites were counted, pelleted by centrifugation (15 min at 3500 rpm) then resuspended at a cell density of 1 to 5×10^8^ cells/ml in buffer A. NP40 was then added (1.2 %), the cells were dounced 30 times with a manual douncer and the suspension centrifuged for 4 min at 14,000 rpm. The soluble fraction (S14) was recovered and dialysed over night (O.N.) against phosphate buffered saline (PBS).

### Serum immunoglobulins

Total immunoglobulins in the sera, and specific Abs against the parasite, were determined by ELISA, as described elsewhere [22], using flat-bottomed plates pre-coated with goat anti-mouse immunoglobulins or 10 µg/ml of *T. vivax* S14 extract [23]. Total IgG or IgM concentrations were deduced from standard curves constructed using purified mouse immunoglobulins or presented as titres.

### Flow cytometry

Spleen and peripheral blood cell (PBL) suspensions (10^7^ cels/ml) were stained with monoclonal Abs diluted in balanced salt solution containing 1% fetal calf serum and 0.01% azide. PBL cells were previously treated with ammonium/chloride/potassium buffer (ACK), pH 7.2, to lyse the red cells. Cells (10^6^ cells) were preincubated with anti-CD16/CD32 (clone 2.4G2) Abs in order to block immunoglobulin nonspecific binding through Fc receptors. Cells were stained with directly-labeled (FITC, PE ou Alexa - Fluor® 647) or biotinylated Abs for 30 minutes on ice. The following Abs were used: CD3 (clone 145.2C11), CD4 (clone GK 1.5), CD5 (clone 53–7.3), CD8 (clone 53–6.7), B220/CD45R (clone RA3-6B2), μchain (clone R6-60.2), Mac 1 (clone M1/70), IgD (clone 11–26), CD19 (clone 1D3), Vβ5.1+5.2 (clone MR9-4), Vβ6 (clone 44.22.1), Vβ8.1+8.2+8.3 (clone F23.1) and Vβ14 (clone 14–2). After washings, biotinylated Abs were further incubated with fluorescein isothiocyanate-streptavidin or phycoerythrin-streptavidin conjugates. Two-color acquisition was carried out with a FACS Scan cytofluorometer (Becton Dickinson) or with a FACScanto (BD biosciences). Dead cells were excluded from the analysis by gating out propidium iodide-stained cells. Splenic and PBL lymphocytes were gated on forward-light scatter/side-light scatter combined gate, and 20000–100000 events were acquired. Number of cycling lymphocytes (S+G2 and M) in the spleen was estimated by measuring individual cell ploidy by dual parameter FSC/SSC combined FACS analysis of cells stained with propidium iodide. Bone marrow-derived cells from two femurs/mouse were recovered in HBSS/2% FCS, counted, rinsed and resuspended at 1×10^8^ cells/ml. The following Abs from PharMingen (coupled with FITC, PE, allophycocyanin (APC), or PE-Cy7) were used: CD3 (clone 145-2C11), Gr1 (clone RB6-8C5), TER-119 (Ly76), CD45R/B220 (clone RA3-6B2), CD19 (clone 1D3), CD11c (clone HL3), NK1.1 (PK136), Mac 1 (clone M1/70), CD117/cKit (2B8), Sca1 (clone E13-161.7), CD34 (RAM34), and CD16/32 (clone 2.4G2). The lineage mix consisted of CD3, Gr1, TER-119, B220/CD45R, CD19, CD11c, NK1.1 and Mac 1 Abs coupled to PE. Staining was performed on 5×10^6^ cells for 30 minutes at 4°C. The cells were then rinsed in HBSS/FCS 2%, fixed for 10 minutes in 2% paraformaldehyde, rinsed in HBSS/2% FCS and resuspended in 400 µl of HBSS/2% FCS. Flow cytometry acquisition of at least 100000–150000 events was performed in a FACSCanto. Results were analyzed by FlowJo software (Tree Star, Inc). Prism software (GraphPad, San Diego, CA) was used for statistical analyses. Intergroup comparisons were made by an unpaired *t* test.

### Histology and immunohistochemistry

Spleens were removed from control and infected mice 20 days post-infection (d.p.i.). Mice were initially anesthetized by an intraperitoneal injection of 0.1 ml per 10 g mouse body weight of a solution containing 1 mg/ml xylazine (Rompun 2%, Bayer, Leverkusen, Germany) and 10 mg/ml ketamine (Imalgène 1000, Merial, Lyon, France) and were then sacrificed by cervical dislocation. After a complete post-mortem examination, the spleen, liver, kidneys, lung, heart and specimens of the central nervous system were removed and immediately fixed in RCL2-CS100 (38%), a non-toxic, formalin-free fixative (Alphelys, Plaisir, France). Tissue samples from these organs were embedded in paraffin and five-micrometer sections were cut and stained with hematoxylin and eosin (HE). The phenotypic profile of the inflammatory infiltrates was determined by immunohistochemical analysis using the following primary Ab, diluted in sterile PBS (VWR, Strasbourg, France) and incubated O.N. at 4°C: rabbit polyclonal anti-human CD3 Ab to detect T lymphocytes (Dako, Glostrup, Denmark), rat monoclonal anti-murine B220/CD45R mAb (clone RA3-6B2) to detect B lymphocytes (Caltag, Burlingame, CA, USA), rat anti-murine F4/80 mAb to detect macrophages (clone BM8, Caltag), and rat anti-murine Ly-49G2 mAb to detect NK cells (clone 4D11, BD Pharmingen, San Diego, CA, USA). After removing the paraffin (xylene followed by ethanol), the slides were treated for 20 min with a blocking solution containing 2% bovine serum albumin diluted in PBS, prior to primary Ab incubation. Primary Ab against B220/CD45R, F4/80 and Ly49G2 were visualized using a Histofine Simple Stain MAX-PO kit (Histofine Biosciences inc, Cambridge, UK) and primary Ab against CD3 were visualized using peroxidase-labeled polymer for rabbit polyclonal Ab (EnVision, Dako, Carpinteria, CA, USA), according to the manufacturer's protocol. Color was developed with 3-Amino-9-EthylCarbazole (AEC chromogen; BD Pharmingen). The sections were then counterstained with Meyer's hematoxylin, and cover-slipped for microscopic examination. Red areas were considered to be positive, according to manufacturer's indications.

### Statistical analyses

All the experiments were performed three or four times using at least 4–5 mice per time point and per experimental group. Mice were analyzed individually and the differences between the groups used in this study were tested for statistical significance using Student's t test whenever appropriate (Prism software, GraphPad, San Diego, CA). The data are expressed individually or as arithmetic means +/− the standard deviation (SD) of the means.

## Results

### *T. vivax* infection induces major modifications in the lymphocyte populations

**Figure 1:**
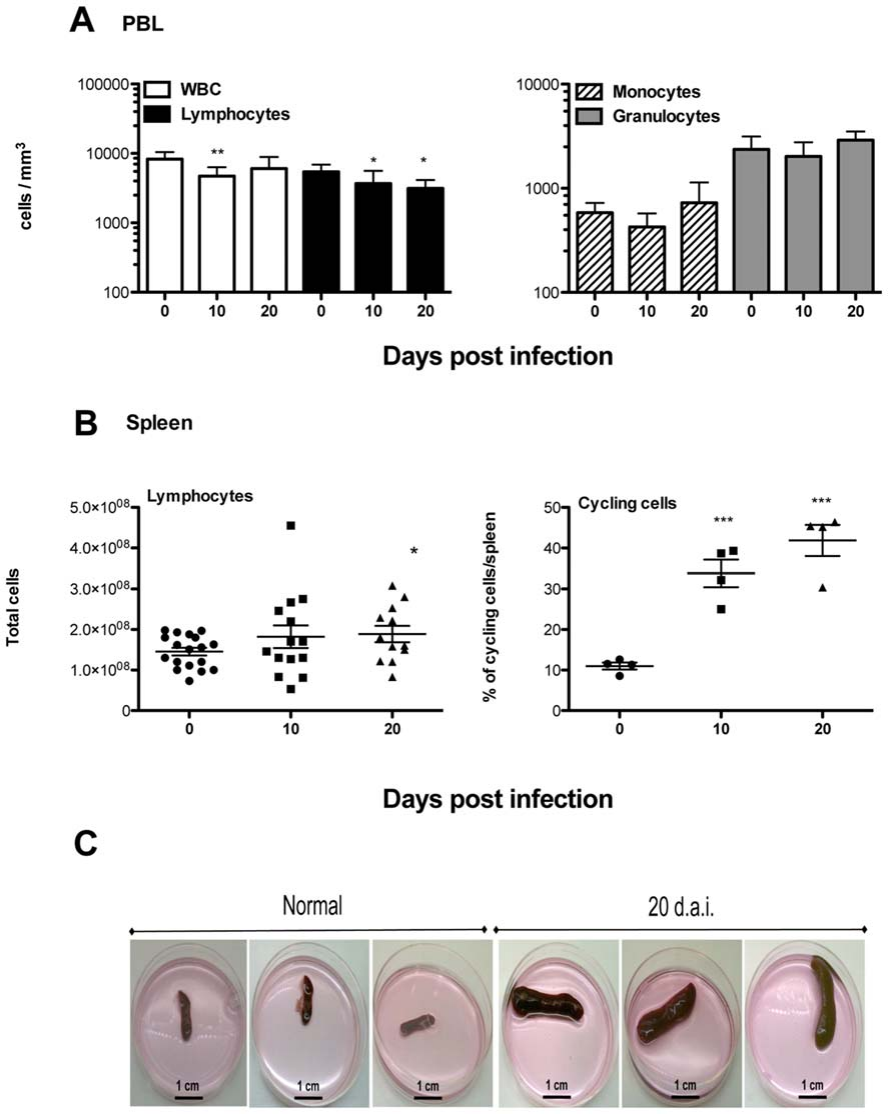
*T. vivax* induces severe splenomegaly as a result of intense blastogenesis. Four to five 8-week-old outbred mice were analyzed per time point (0, 10 and 20 d.p.i.) and per experiment. Mice were infected i.p. with 10^2^ bloodstream forms of *T. vivax* and compared to control age-matched uninfected controls. Numbers of peripheral white blood cells (WBC), lymphocytes (A, left panel), monocytes and granulocytes (A, right panel) were determined individually, as indicated in the methods section, throughout the ensuing infection and expressed as means +/− SD of the means. Spleen cells were recovered at different time points and total numbers of lymphocytes were depicted individually given the variable number of cells obtained from individual outbred mouse used in 4 different groups of experiments (B, left panel); frequencies of cycling cells were also determined individually in one out of four experiments (B, right panel). Arithmetic means and SD of the means are presented. * p<0,020; ** p<0,007; *** p<0,0002 when compared with samples from day 0. Macroscopic examination of spleens harvested from three uninfected (normal) and three infected (20 d.p.i.) outbred mice showing the marked splenomegaly; scale bars = 1 cm (C).

Classic features of *T. vivax* trypanosomosis such as severe acute anemia and the remodeled secondary organs were observed in our recently developed experimental murine models of *T. vivax* infection (see accompanying paper). In the present study, whereas lymphocytes and white blood cells decreased significantly in the first 10 days of infection (Figure 1A, left panel), monocytes and granulocytes numbers were not significantly altered by the infection (Figure 1B, right panel). In the same manner as with other trypanosome infections in mice and cattle, *T. vivax* triggers marked lymphocyte blastogenesis that swells cell numbers in secondary lymphoid organs. In fact, as can be seen in Figure 1B, the number of lymphocytes increased throughout the infection (left panel), reflecting the stimulation of the immunological apparatus. We noted that the spleen enlargement observed was not due only to an increase in cell numbers (1.5 fold) but also to the elevated number of cycling cells (Figure 1B, right panel) in the organ. Also, as compared to spleens from uninfected mice, those from infected individuals included a large number of dead cells (2–3 fold the number of live cells) that were appropriately excluded from the present analyses. As a result, despite considerable individual differences in cell numbers expressed by the mice used in those studies, spleen enlargement is evident as early as 10 d.p.i. and is massive at 20 d.p.i., constituting splenomegaly (Figure 1C).

### *T. vivax* infection is associated with severe B lymphocyte depletion in the spleen

**Figure 2:**
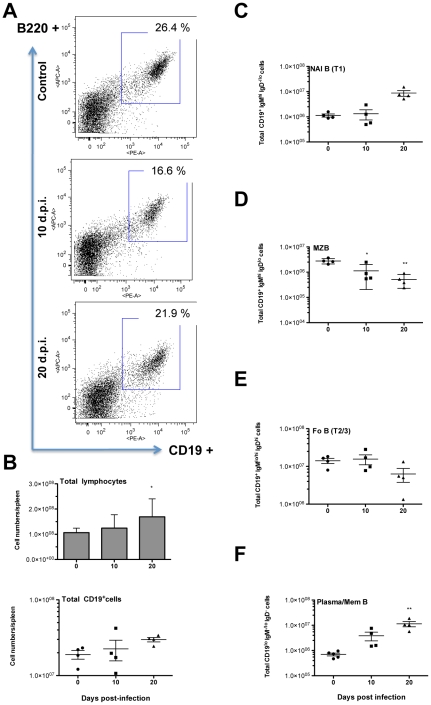
Splenic B lymphocyte populations fall dramatically following infection. Four to five 8-week-old outbred mice were analyzed per time point (0, 10 and 20 d.p.i.). Mice were infected i.p. with 10^2^ bloodstream forms of *T. vivax* and spleen cells were recovered 10 and 20 days post infection for comparison with those recovered from age-matched normal uninfected controls. Spleen cells were stained by immunofluorescence with specific Abs directed against CD45R/B220 (B220) and CD19. Two-color acquisition was carried out on a FACS Scan cytofluorometer and the results analyzed using FlowJo software. The figure depicts the results of a representative experiment out of three using the similar number of individuals. SD of the means are presented. * p<0,038; ** p<0,003 when compared with samples from day 0. The dot plots illustrate the FACS distribution of mouse spleen cells according to the expression of CD19 and B220 at 10 d.p.i and 20 d.p.i., as compared to cells from normal uninfected mice; similar results were obtained in different mice drawn from the same group and analyzed individually (A). Arithmetic means of total lymphocyte numbers (upper panel) or individual CD19^+^ cell counts (lower panel) are depicted +/− standard deviation of the means (B). CD19^+^ cells were gated and distributed in double plots for the expression of IgM and IgD (*see gating strategy in Figure S1*). Numbers of Newly Arrived Immature B cells (CD19^+^IgM^hi^IgD^−/lo^, NAI B-T1) (C), Marginal Zone B cells (CD19^+^IgM^hi^IgD^lo^, MZB) (D), Follicular B cells (CD19^+^IgM^lo/hi^IgD^hi^, Fo B) (E), and plasma/memory cells (CD19^+/lo^IgM^−^IgD^−^, Plasma/Mem B) are shown in (F).

To investigate whether the increase in cell numbers described above involves all lymphocyte populations or is restricted to certain subsets, spleens were isolated at different time points during the infection and cell suspensions prepared for characterization by flow cytometry. Archetypal CD3^+^CD4^+^, CD3^+^CD8^+^, B220^+^IgM^+^ and CD5^+^IgM^+^ B1 populations were analyzed (Table 1). Only days 10 d.p.i. and 20 d.p.i., which correspond respectively to peak of parasitemia in outbred mice and to the day that generally precedes death were shown as compared to normal age-matched uninfected controls (*see Material and Methods*). Firstly, while the total number of spleen cells increased up to 1.5 fold, the total number of CD3^+^CD5^+^ lymphocytes increased 1.8–2 fold at 10 d.p.i.. More precisely, while the CD4/CD8 ratio appeared to be unaffected in the first 10 days of infection (2.4∶1), a fall in CD8^+^ cells and an expansion of the CD3^+^CD4^+^ subset was observed from 10 d.p.i., significantly increasing the CD4/CD8 ratio (6∶1). Furthermore, and similarly to what has been described elsewhere for other trypanosoma infections double negative CD4^−^CD8^−^ (possibly gamma-delta T cells) [24]–[25] cells increased 5 fold at 10 d.p.i. and accounted for almost 10% of the increase in CD3^+^CD5^+^ cell numbers (Table 1). When the expression of major TCR-Vβ chains by CD3^+^CD5^+^ cells was investigated, this failed to show any preferential growth of a particular T-cell population but rather reflected the polyclonal nature of the expansion in the T cell repertoire. At the same time, a progressive significant decrease in the number of B220^+^IgM^+^ B cells was pointed out during the course of infection. Similarly to previous data obtained in cattle and sheep infected with other trypanosomes [26], [27], this experimental *T. vivax* infection stimulated the expansion of CD5^+/lo^IgM^hi^ B cells (B1 cells) in the first 10 d.p.i. but a return to normal levels thereafter. Nevertheless, together with the increases observed in the CD3^+^CD4^+^ cell count, this commonly substantiates the B/T cell ratio inversion observed as early as 10 d.p.i.. The frequency of B220^+^CD19^+^ cells in the spleen was determined during the infection using additional experimental groups of mice and corroborated the consistent progressive fall in the frequencies of splenic B cells (see Figure 2A for one exemple). However, since lymphocyte cell numbers in the spleen increased throughout infection (∼1.5 fold) total counts of B cells were not systematically and significantly decreased if evaluated by the corresponding relative numbers of B220^+^CD19^+^ (Figure 2B). This seems to be the consequence of both the individual mouse differences in CD19 cell frequencies and the variability of total spleen cell numbers disclosed by the mice in different experimental groups. Nonetheless, close examination within CD19^+^ gated population revealed dynamic and reliable alterations of B cell subpopulations in the spleen, as can be evalluated by the separate representation of mouse data (*see Figure S1 for the gating strategy*). Therefore, newly arrived immature B (NAI B) cells increase in the organ (Figure 2C). In contrast, a gradual and significant decrease in marginal zone B (MZB) cells and to a lesser extent in follicular B cell counts is observed during the infection (Figures 2D and 2E), as similarly described for *T. brucei* infection [6]. As expected, the number of plasma/memory cells (IgM^−^IgD^−^) considerably increased over time and did not return to normal levels even in the late stages of the infection (Figure 2F), contrasting with data obtained in *T. brucei-*infected mice [6].

**Table 1:** Changes in spleen cell populations.

Cell Type (x 10^6^)	Uninfected	10 d.p.i.	20 d.p.i.
**Total Spleen cells**	145.0±39.9	188.0±55.7 *	215.0±57.1 ***
**CD3^+^ CD5^+^**	62.0±3.1	125.0±53.8 *	138.0±37.0 ***
**CD3^+^ CD4^+^**	54.2±9.9	70.7±40.5	122.0±17.1 *
**CD3^+^ CD8^+^**	29.3±3.3	28.9±9.3	20.3±0.5
**CD3^+^ CD4^−^ CD8^−^**	2.29±1.3	10.1±7.4	13.0±12.3
**CD3^+^ Vβ5^+^**	3.5±0.02	13.9±7.0	11.1±7.5
**CD3^+^ Vβ6^+^**	26.1±3.4	26.3±11.2	29.3±10.8
**CD3^+^ Vβ8^+^**	35.2±12.4	57.1±41.4	41.1±7.21
**CD3^+^ Vβ14^+^**	4.7±2.3	2.7±2.0	8.9±2.4
**B220^+^ IgM^+^**	74.3±12.3	60.8±36.5	42.8±13.3 *
**CD5^lo^IgM^+^ (B1)**	5.7±2.0	17.1±9.5	7.5±3.5

Arithmetic means ± SD of the means of two independent experiments performed with at least 3–5 mice per group analyzed individually. * p<0.0360, *** p<0.0004 when compared with means from day 0.

**Figure 3:**
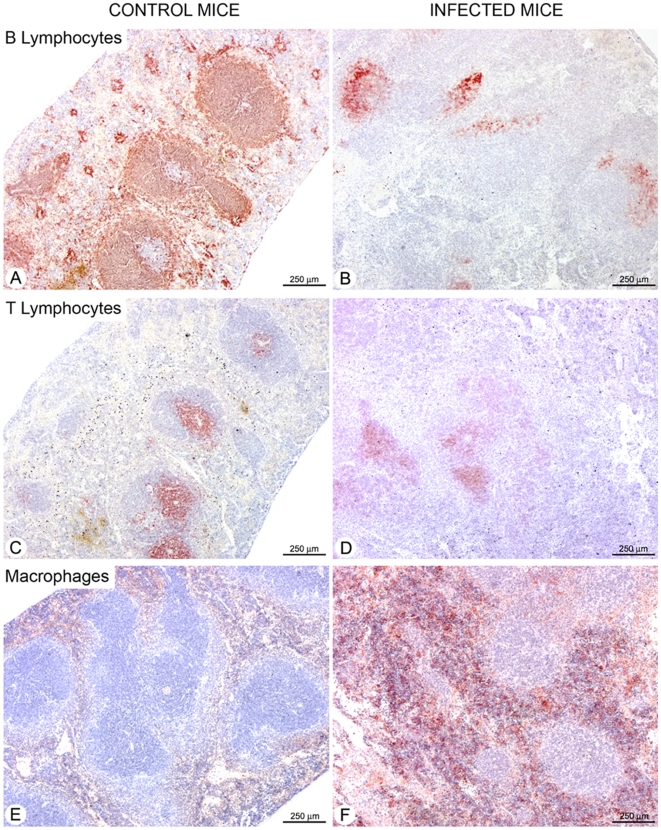
Loss of splenic B lymphocytes is associated with marked macrophage infiltration. Serial sections of spleens from uninfected controls (A, C and E) and from mice infected with 10^2^ bloodstream forms of *T. vivax* 20 d. p. i. (B, D and F) were fixed and further stained with B220 (A, B), anti-CD3 (C, D) or anti-F4/80 (E, F) to detect B lymphocytes, T lymphocytes and macrophages, respectively. The sections are representative of 5 mice analyzed individually per time point. For information, frequencies of gated splenic macrophages using specific Mac-1 antibodies obtained by flow cytometry using an enlarged forward scatter/side scatter combined gate of spleen cell suspensions corresponded to 7.6% and 6,75% respectively, for days 10 and 20 of infection, as compared to 2,29% obtained from normal uninfected mice.

In order to gain further insight into spleen cell dynamics, we set about analyzing the functional architecture of the spleen by *in situ* immunohistochemistry. As can be seen in Figure 3, B lymphocytes were found exclusively in the spleen white pulp of control mice, more specifically in the lymphoid follicles (Figure 3A). By contrast, the spleens of infected mice (Figure 3B) showed a markedly disorganized white pulp associated with severely depleted B cells in the follicles. Major differences in T lymphocyte distribution were also observed between the control and infected animals (Figures 3C and 3D), most probably corresponding to the intense tissue disorder resulting from substantial cellular infiltration, conspicuously noted for macrophages in the red pulp of infected spleens (Figures 3E and 3F).

### *T. vivax* infection triggers intense hematopoiesis and bone marrow population dynamics

**Figure 4:**
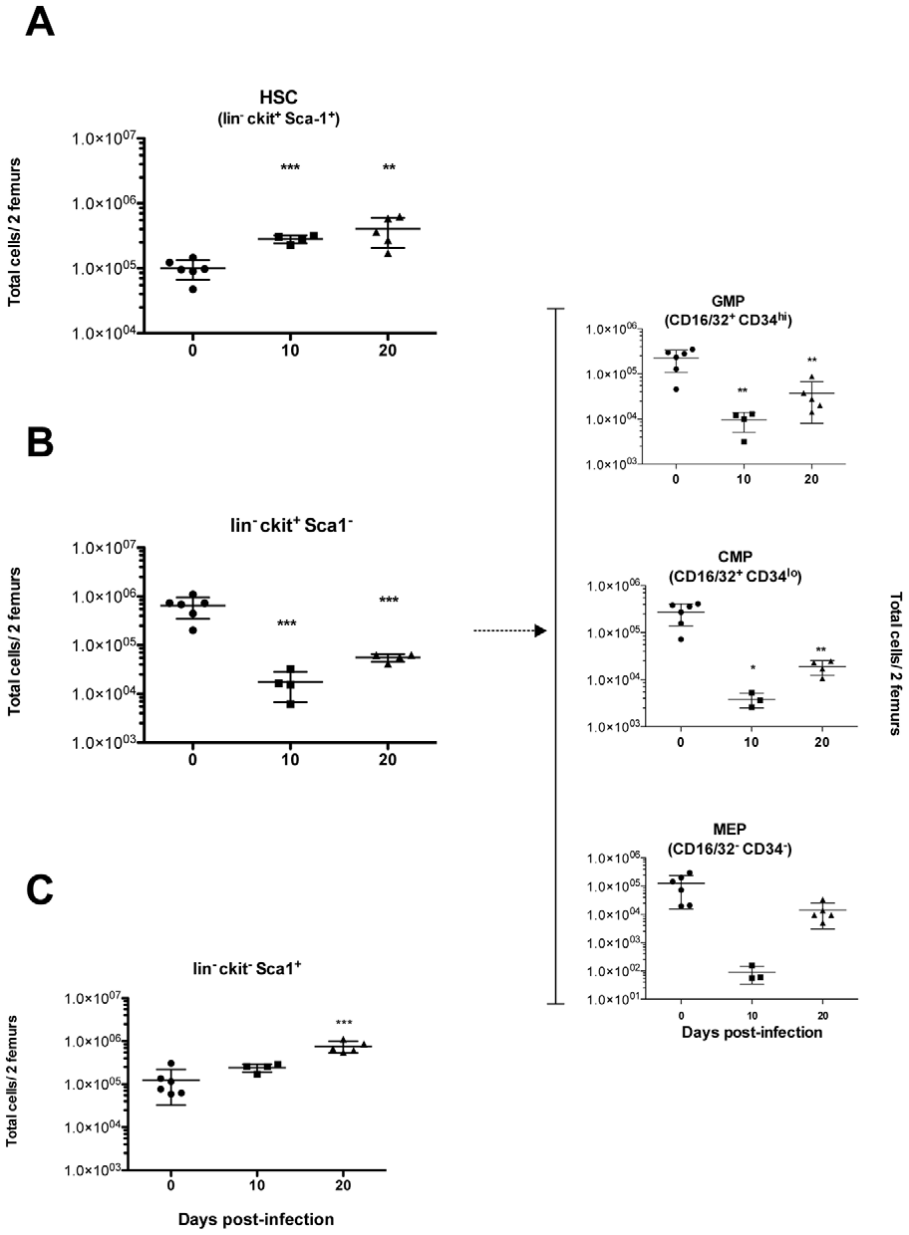
*T. vivax* infection induces increased hematopoiesis but a collapse in granulocyte/monocyte, common myeloid and megakaryocyte precursors. Mice were infected i.p. with 10^2^ bloodstream forms of *T. vivax*. Pool of bone marrow cells were obtained from both femurs of three to six 8-week-old outbred mice then analyzed per animal at 10 and 20 d.p.i. by immunofluorescence and compared with bone marrow cells from normal age-matched uninfected controls. FSC-A/SSC-A and FSC-W/FSC-H combined plots were used to gate total bone marrow cells and to eliminate doublets and debris (*for gating strategy, see Figure S2*). Increases in the number of hematopoietic stem cells (HSC) during the infection (cKit^hi^Sca1^+^) within the “lineage negative” (lin^−^) gated population, are depicted in (A). Granulocyte-monocyte Precursors (GMP, CD16/32^+^CD34^hi^), Multipotent Common Myeloid Precursors (CMP, CD16/32^+^CD34^lo^) and Megakaryocytes and Erythroblasts Precursors (MEP, CD16/32^−^CD34^−^) (B, right panel), were obtained from gated lin^−^cKit^+^Sca1^−^ cells (B, left panel), distributed in double plots for CD16/32 and CD34 markers. Lin^−^cKit^−^Sca1^+^ cell numbers are shown (C). Results are expressed per individual mouse and are representative of at least 2 different experiments per time point. Arithmetic means ± SD of the means are presented. * p<0.01, ** p<0.005, *** p<0.0001 when compared with samples from day 0.

The effect of *T. vivax* infection on the number of bone marrow cell precursors was studied in an attempt to identify potential hematopoietic abnormalities that could explain the substantial decrease in the peripheral counts of B cell populations. Mice were infected and bone marrow cells recovered and analyzed by flow cytometry 10 and 20 days post infection (*see* *Materials and Methods* *and Figure S2 for gating strategies*). The frequency of hematopoietic stem cells (HSC) and early progenitors was then determined within the gated lineage^−/lo^ (lin^−/lo^) bone marrow cell fraction that did not express (or expressed only low levels of) markers for mature cells. Thus, Figure 4A shows that while untreated controls possessed a quiescent number of HSCs, the bone marrow was strikingly enriched with pluripotent HSCs (lin^−/lo^ cKit^hi^ Sca1^+^) in infected mice. While the total number of bone marrow cells obtained from two femurs did not increase significantly, HSC numbers increased twice as early as 7 d.p.i. (not shown) and remained high throughout the infection (up to 5 fold increase by 20 d.p.i.). Most of these HSCs had only a short-term capacity for reconstitution since more than 80% expressed CD34 reflecting a marked switch from the G0 state to an active cell cycle (data not shown). It is worth noting that HSC numbers deduced from the lin^−/lo^ cKit^+^Sca1^+^ gate include a third of cells expressing low levels both cKit and Sca-1. This lin^−/lo^cKit^lo^Sca1^lo^ fraction comprises Commun Lymphoid Progenitors (CLP), gives rise to all lymphoid lineages and differentiates from HSC after up-regulation of IL-7R (not determined here). As a result, the marked increase in HSC/CLP combined population, reflects the intense hematopoiesis triggered by the infection. It is important to note the marked decreases observed in lin^−^ckit^+^Sca1^−^ marrow cell populations over the first 10 days of the infection that persisted over time (Figure 4B). The distribution of these cells in relation to the expression of CD16/32 and CD34 cell markers (Figure 4B, right panels), showed that the frequencies of Granulocyte-Monocyte Precursors (GMP, CD16/32^+^CD34^hi^) and Common Myeloid Progenitors (CMP, CD16/32^+^CD34^lo^) were significantly altered by the infection as compared to uninfected controls. It is interesting to note that although Megakaryocyte Erythrocyte Precursor numbers (MEP, CD16/32^−^CD34^−^) decreased upon infection (see Figure 1B, right panel), this decrease only became statistically significant on day 21 of the infection, just before death (not shown) corroborating the previously observed thrombocytopenia (see accompanying paper and [10], [28]). Interestingly, the highly reconstituting cells presenting the lin^−^cKit^−^Sca1^+/hi^ phenotype [29] increased substantially in number during the infection (Figure 4C) and peaked on day 20.

### Peripheral B lymphocyte exhaustion is associated with a defect in B cell development

**Figure 5:**
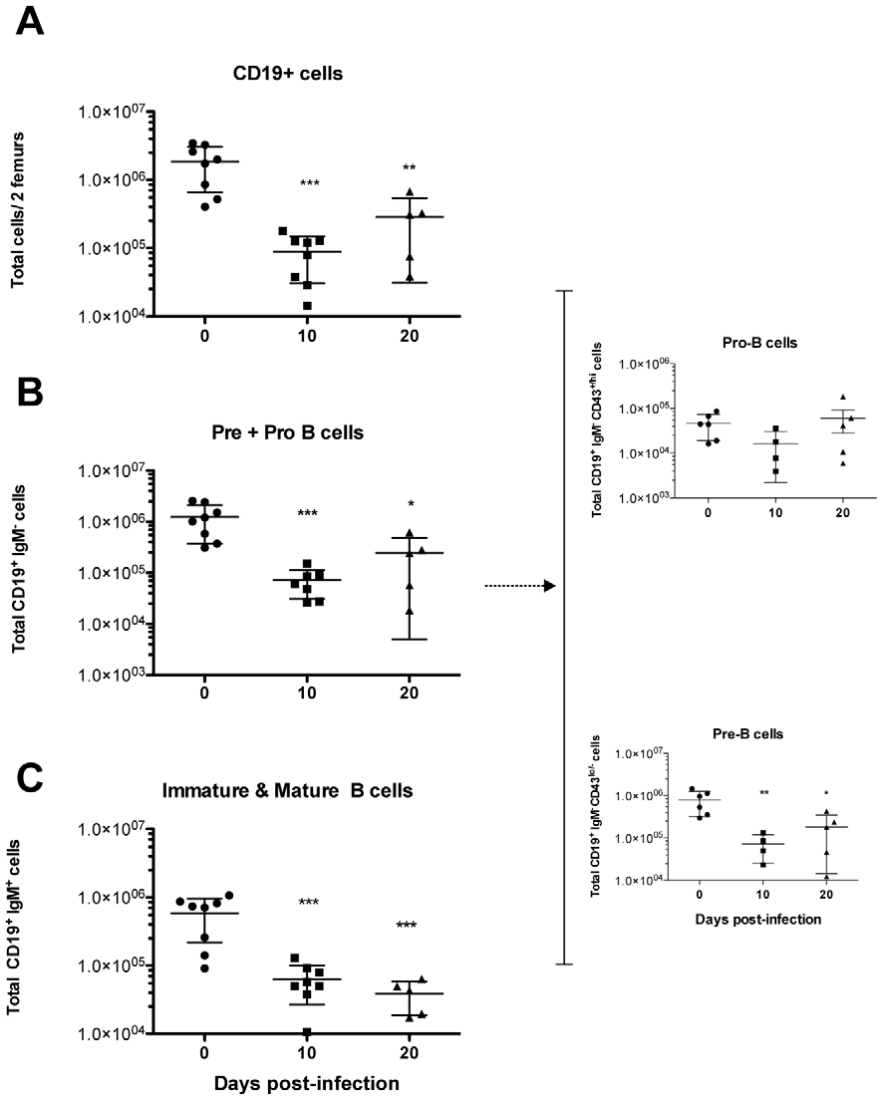
*T. vivax* infection leads to elevated bone marrow dynamics and alterations in the maturation of B-cell progenitors. Mice were infected i.p. with 10^2^ bloodstream forms of *T. vivax*. Pools of bone marrow cells were obtained from both femurs of four to eight 8-week-old outbred mice then analyzed per animal at 10 and 20 d.p.i. by immunofluorescence and compared with bone marrow cells from normal age-matched uninfected controls. Cells were stained for IgM, CD19 and CD43, and SSC-A/FCS-A combined plots were used to gate lymphocyte populations. Doublets were eliminated by a FSC-W/FSC-H combined gate (*see Figure S3, for gating strategy*). CD19^+^ cells were gated and total numbers of positive cells per 2 femurs are depicted in (A); PreB + Pro B (CD19^+^IgM^−^) and late immature/mature B (CD19^+^IgM^+^) cell numbers are shown respectively in (B) and (C); total numbers of Pro-B and Pre-B cells (B, right panel) were deduced from the expression of CD43 by CD19^+^IgM^−^ Pre B + Pro B gated cells. Results are expressed per individual mouse and are representative of at least 3 different experiments per time point. Arithmetic means ± SD of the means are presented. * p<0.05, ** p<0.001, *** p<0.0001 cell when compared with samples from day 0.

We next analyzed bone marrow cell populations committed to the B cell lineages. As can be seen in Figure 5A CD19^+^ cell counts decreased significantly throughout the course of infection. Similarly, the number of Pre B + Pro B (CD19^+^IgM^−^) cell progenitors in the CD19^+^ gated population decreased more than 10 fold during the course of infection (Figure 5B; *see Figure S3 for gating strategy*). It is noteworthy that marked individual differences, but statistically significant, were observed for CD19^+^IgM^−^ at late stages of the infection (i.e. day 20), when generally only 40% of the infected individuals are still alive (see accompanying paper). Some fluctuations were observed in the number of immature/mature B cells (CD19^+^IgM^+^) from day 7 of infection (not shown). This cell population then significantly decreased 10 d.p.i. at the peak of parasitemia and thereafter (Figure 5C, and accompanying paper). These findings are consistent with the emigration of these immature/mature B cells to reconstitute the peripheral B cell pool. However, after 10 days of infection, while more than 70% of B cells expressed IgM, immature pro-B progenitors (CD19^+/lo^IgM^−^CD43^+/hi^) were seen to grow in number as the infection progressed, but their differentiation into pre-B cells appeared to be delayed since no proportional increase was observed in CD19^+^IgM^−^CD43^−/lo^ (Figure 5B, right panel). Altogether, these results suggest that the process of B cell hematopoiesis is disturbed in the bone marrow and may be instrumental in the subsequently diminishing production of mature B cells (CD19^+^IgM^+^CD43^−^) in the periphery.

### The massive hypergammaglobulinemia induced by the infection is mainly composed of antibodies that do not recognize parasite antigens

**Figure 6:**
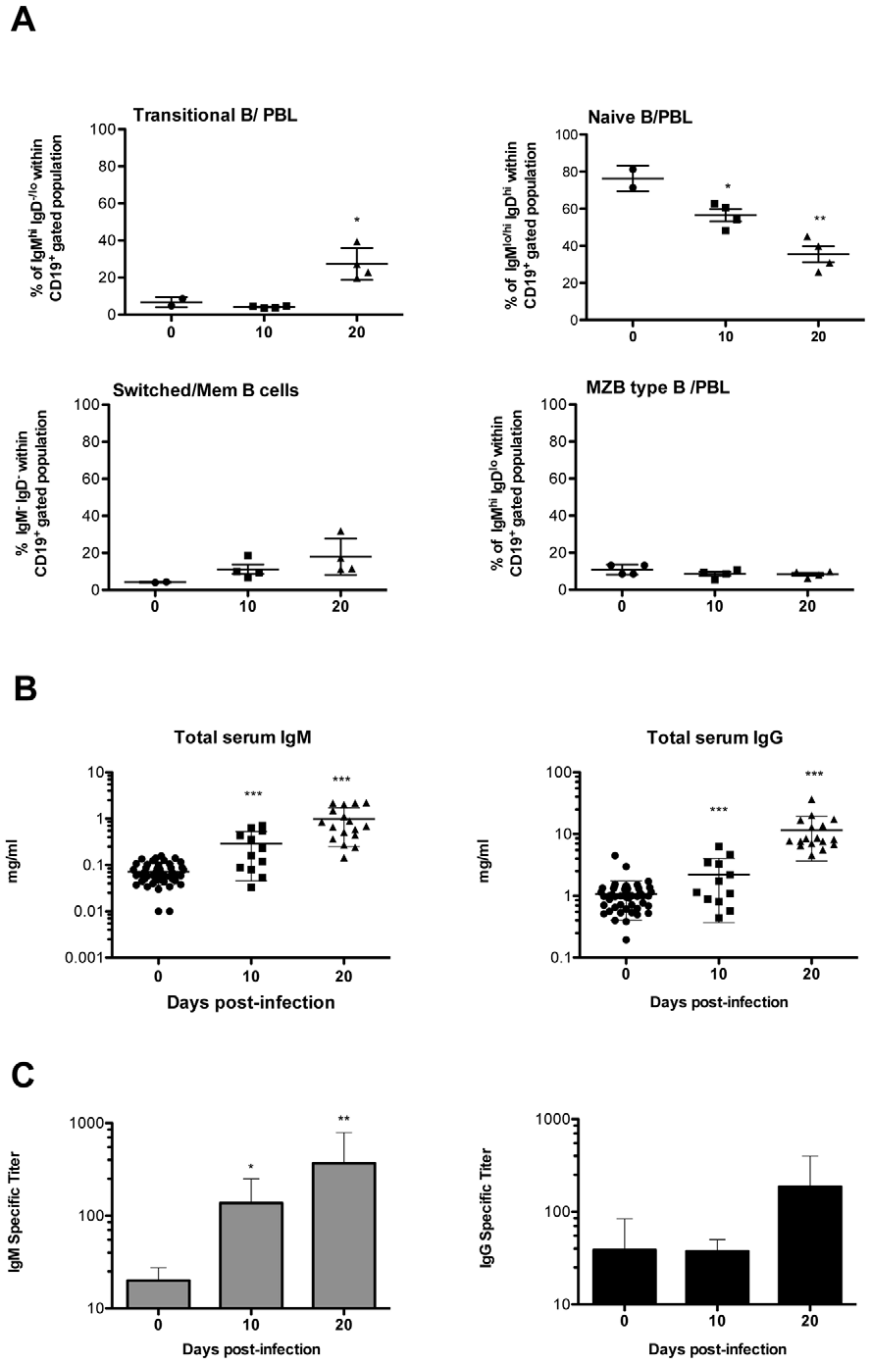
*T. vivax* infection results in massive production of non-specific IgG. PBL and sera from individual mice were collected at different time points following infection with 10^2^ bloodstream forms of *T. vivax*. PBL cells obtained from 2 uninfected and 4 infected mice per group were subjected to FACS analysis and numbers of recently immigrated “transitional” B cells (IgM^hi^IgD^−/lo^), naïve B cells (CD19^+^IgM^lo/hi^IgD^hi^), and Post germinative center “switched” plasma/memory B cells (CD19^lo^IgM^−^IgD^−^) determined per individual mouse (A) within the CD19^+^ gated population (*for gating strategy see Figure S4*). Total circulating IgM and IgG immunoglobulins (B) and parasite-specific IgM and IgG titers were determined individually (C). Results in B and C were obtained from at least 12 different mice per time point from different experiments. For information, experiments performed with C57BL/6 mice showed similar amounts of total IgG (20 mg/ml by day 20) but ten fold higher quantities of total IgM (10 mg/ml by day 20), but similarly low titers of parasite specific IgG and IgM responses were observed (1/300 an 1/100, respectively). Arithmetic means ± SD of the means are presented. * p<0.05, ** p<0.001, *** p<0.0001 when compared with samples from day 0.

A study made of CD19^+^ PBL B cells in relation to their expression of IgM and IgD detected several sub-populations of circulating B cells (*see Figure S4 for gating strategy*). Thus, late in the infection, the number of cells recently immigrated from the bone marrow (Transitional B) increases in parallel with the fall in numbers of B cells in lymphoid organs (Figure 6A). It was also noted that the number of naïve B lymphocytes decreased considerably throughout the study period analyzed, contrasting with the relative rise of “switched” plasma/memory cells (IgM^−^IgD^−^). In order to determine the impact of the decreased B lymphocyte repertoire on the production of immunoglobulins, total IgM and IgG serum levels were quantitated during the infection and their ability to recognize parasite antigens was determined. Sera obtained from the infected mice were tested individually for their ability to react with total *T. vivax* extracts prepared at the peak of parasitemia (10 d.p.i.) given that outbred mice showed a parasitemia plateau after 10 d.p.i. that seems coherent with a parasite population expressing thereafter the same variable antigen (VAT). While IgM and especially IgG increased 5–10 fold on infection (Figure 6B), Ab production only partially correlate with a capability to specifically recognize *T. vivax* antigens (Fig. 6C). As expected, IgM specific antibody titers directed against the parasite were regularly maintained throughout the infection as a result of increasing parasite load or to (newly) produced IgM responses directed to (possibly new) VSGs (1/100 to 1/500 dilution out of 1–5 mg/ml of seric IgM). This observation contrasted with the high levels of non specific polyclonal IgG recorded at the same time period (only 1/25 to 1/100 serum dilution out of 10–20 mg IgG/ml reacts with *T. vivax* extracts). This high production of IgG Abs correlates with the rise in peripheral “switched/memory” (IgM^−^IgD^−^) B cells, possibly engaged with the production of ‘non-IgM’ classes or isotypes of immunoglobulins. These data more than substantiating the polyclonal nature of the B cell response induced by the infection, indicate that *T. vivax* induces a B cell response that is for the most part not directed to the parasite contributing to its evasion strategies.

## Discussion

The work described herein provides evidence that experimental *Trypanosoma vivax* infection induces rapid and marked bone marrow hematopoiesis, and a significant delay in the maturation of B-cell progenitors. Maintenance of the peripheral B cell pool requires the continuous input of newly formed B cells [30]. Although our findings show that mature B cells are recruited into the periphery to replenish the pool of terminally differentiated Ig-secreting cells responsible for the massive hypergammaglobulinemia triggered by the infection, an imbalance in B cell development and supply nevertheless persists. Furthermore, early infection decreases the frequency of downstream B-cell progenitors, consequently reducing the flow of mature B cells into the periphery. It was previously shown that parasite-derived B cell directed apoptotic signals cause severe destruction of the available B cell pool in the spleen [6]. Our present findings show in addition that the infection also disturbs bone marrow maturation dynamics, thereby preventing homeostasis. Moreover, a critical drop of Granulocyte-Monocyte and Common Myeloid follows the infection. In addition, the significant decrease of Megakaryocyte Erythrocyte Precursors supports the thrombocytopenia observed with the onset of parasitemia (accompanying paper).

As expected, the infection triggers rapid and persistent bone marrow activity leading to the production of high levels of long-term reconstituting stem cells. HSC activation is accompanied by significant bone marrow dynamics but not by the appropriate differentiation of progenitors committed to both myeloid and lymphoid lineages. Rather, the infection induces the expression of Sca-1 in a considerable number of ckit^−^ hematopoietic progenitors, possibly resulting from the release of pro-inflammatory cytokines, as postulated [29], [31]. It is also possible that CpG-DNA motifs released from the parasite may trigger the production of IFN type I, lead to the intense activation of B cells and equally contribute to the expression of Sca-1 by bone marrow cells [32]–[34]. These recently described ckit^−^Sca-1^+^ bone marrow cells [29] possess the potential to more rapidly generate B and T cells than CLPs. Therefore, it is possible that a time lag in the maturation of lymphoid and myeloid precursors is observed as a consequence of differential ontogenesis. Although speculative, one hypothesis would consider that parasite-derived mitogenic (or superantigenic) effect on B cells at the periphery could bring about a compensatory effect in the bone marrow to reestablish homeostasis. However, our results indicate a deficiency (or developmental arrest) in B-cell precursors further impacting on the availability of mature B cells at the periphery and altering the B: T cell ratio in lymphoid organs.

Mouse infection with *T. brucei* results in profound spleen remodeling with a dramatic drop in IgM^+^ marginal zone B cells (MZB) due to specific apoptosis [6]. Our studies provide further evidence that the mouse infection with *T. vivax* causes a marked reduction in the spleen of both B220^+^CD19^+^IgM^+/hi^IgD^lo^ (MZB) and B220^+^CD19^+^IgM^lo/hi^IgD^hi^ follicular B cells. It would thus appear that the splenomegaly and the increase in cellularity observed result from intense, compensatory T-cell blastogenesis and a significant infiltrative process, mostly composed of macrophages. These findings are supported by the fact that GMP bone marrow numbers decrease by the second week of infection, compatible with the further mobilization of macrophage/monocyte cells to committed organs, as confirmed by histopathological studies. Since no bone marrow atrophy is seen during *T. vivax* infection, several different hypotheses may be advanced to explain the recurring waves of activation/repression that affect some specific bone marrow-derived progenitors and the consequent delay in B cell development which impacts on the release of B cells to the periphery. One hypothesis considers the possible down regulation of c-kit expression by marrow cells that are unable to respond to stromal cell factors, thus compromising the generation of new lineage progenitors [35]. However, this is unlikely given that lin^−^ckit^hi^ increase in number in the course of the infection. Instead, a deficiency in IL-7 (or IL-7R) and IL-12, which greatly commits HSC into lymphopoiesis, or an increase in IL-15 which interferes more specifically with pre-B cell proliferation, may impair the development and maturation of bone marrow-derived progenitors [36]–[39]. For instance, it was previously reported that increases in TGFβcause down regulation of both IL-7 [40] and VCAM-1 gene expression [41] by stromal cells, interfering with the self-renewal and differentiation of B-cell precursors. However, only a few studies concerning the differential regulation of cytokine expression in mice that are more susceptible or more tolerant to *T. brucei* and *T. congolense* infections are currently available to further substantiate these hypotheses [42]–[45]. Interestingly, recent reports have attributed the disappearance of peripheral blood leukocytes to a parasite released factor that, through CD45, manipulates the host's cytokinic and adaptive responses, inducing lymphotoxicity [7], [46].

Major dysfunctions in the development of bone marrow cell lineages and greatly impacting on the availability of peripheral lymphoid repertoires have previously been described in other infection models, especially those using MCMV and LCMV viruses [47]–[49]. These abnormalities were shown to be associated with the inability of B- and T-cell subsets to respond to homologous and heterologous antigens, characterizing nonspecific polyclonal lymphocyte responses and the immunosuppression that invariably follows infectious processes [18]. In agreement with the results obtained in livestock and in experimental trypanosome infections, and more specifically with *T. vivax*-infected cattle [18], [50]–[52], we show here that the infection in the mouse experimental model also induces non-specific (but microorganism-triggered) polyclonal B cell responses. This is worsened by the presence in the spleen of numerous abnormal plasmocytes (Mott cells) that are defective in the production of immunoglobulins (see accompanying paper). The presence of Mott cells in the plasma and their relationship with the failure of individuals infected with African trypanosomes to mount efficient B cell responses, has previously been described [53]. It is possible that MZB cells emigrating from the spleen red pulp differentiate into short-lived plasma cells that would mainly produce T-independent, non parasite-directed, B cells responses. However, the rapid decay of MZB cells is rather compatible with these cells playing a role in the capture of new VSG expressed by the parasite during the infection and their subsequent transport into the splenic follicules for antigen presentation to follicular B cells. This hypothesis seems consistent with our present and other previous data obtained with *T. brucei* infected mice [6], where similar decreases of follicular B cells as the infection progresses parallels the increase of IgM-producing (parasite-specific) plasma cells at the periphery. The inability of infected individuals to produce long-lasting amounts of antigen-specific IgG Abs and the levels of antigenic variation displayed by the parasites impose major difficulties that prevent immune intervention against trypanosomosis. The experimental model described here may stimulate further studies on B cell development and fate following *T. vivax* infection and contribute to unraveling pathways of the host-parasite interaction that could help in the design of new therapies for disease control.

## Supporting Information

Figure S1 Gating strategy for the analysis of B cell populations in the spleen. Spleen cells were stained with CD19, IgM and IgD antibodies. 105 events were acquired in a FACScanto (BD biosciences). Lymphocytes were analyzed inside a combined FCS/SSC gate. CD19^+^ cells were gated and distributed in double plots for IgD and IgM expression. Frequencies of the different B cell populations were determined by the differences in the expression of these markers inside the gated population, as follows: Newly arrived immature B cells (IgMhiIgD-/lo, NAI B); Marginal Zone B cells (IgMhiIgDlo, MZB); Follicular B cells (IgMlo/hiIgDhi, Fo B) and Plasma/memory B cells (IgM-/loIgD-, Plasm/Mem B). The figure shows examples of plots obtained with a normal, uninfected mouse or 20 d.p.i. mouse inoculated with 102 bloodstream forms of *T. vivax*.
(0.38 MB TIF)

Figure S2 Gating strategy for the analysis of bone marrow cell lineages and progenitors. Bone marrow (BM) cells obtained from 2 femurs were stained with a combination of antibodies for multi-parameter flow cytometry (see Materials and Methods). Briefly, 100000–150000 events were acquired in a FACScanto (BD biosciences). (A) Combined FSC-A/SSC-A and FSC-H/FSC-W gates were used to gate single BM cells. Cells were then analyzed for the expression of lineage markers (lin). Hematopoietic stem cells (HSC) and early progenitors were gated within the lin-/lo fraction as they do not express, or express low levels of mature cell markers. (B) Frequencies of Lin-/lo BM cells were further analyzed for the expression of Sca1 and c-Kit. LSK (lin-/loSca1+cKit+) fraction contains the HSC and was used to determine the frequencies of short and long reconstituting cells by their expression of CD34 (not shown). Identification of progenitor populations was performed using the lin-/loSca1+cKit+ BM gated cells and their expression of CD16/32 and CD34, as follows: Granulocyte/macrophage progenitors (CD16/32hi/CD34hi, GMP); Common myeloid progenitors (CD16/32lo/CD34hi, CMP) and Magakaryocyte/erythrocyte progenitors (CD16/32-CD34-, MEP). The figure shows examples of plots obtained with a normal, uninfected mouse or 20 d.p.i. mouse inoculated with 102 bloodstream forms of *T. vivax*.
(0.69 MB TIF)

Figure S3 Gating strategy for the analysis of bone marrow cell B cell precursors. Bone marrow (BM) cells obtained from 2 femurs were stained with a combination of antibodies for multi-parameter flow cytometry (see Materials and Methods). Briefly, 100000–150000 events were acquired in a FACScanto (BD biosciences). (A) Combined FSC-A/SSC-A and FSC-H/FSC-W gates were used to restrain the analysis to single BM lymphoid cells. BM cells were further analyzed on the basis of lineage markers (lin). (B) lin+ fraction was then distributed for the expression of CD19. CD19^+^ cells were gated and frequencies of Pre+Pro B cells and late immature/mature B cell populations were identified on the basis of IgM expression inside the gated population. The expression of CD43 by Pre+Pro B gated cells (lin+CD19+IgM-) gave rise to the determination of Pre-B (CD43^−^) and Pro-B (CD43^+^) cell frequencies. The figure shows examples of plots obtained with a normal, uninfected mouse or 20 d.p.i. mouse inoculated with 102 bloodstream forms of *T. vivax*.
(0.48 MB TIF)

Figure S4 Gating strategy for the analysis of of B cell populations in blood. PBL cells were stained with CD19, IgM and IgD antibodies. 20000 events were acquired in a FACScanto (BD biosciences). Lymphocytes were analyzed inside a combined FCS/SSC gate. CD19^+^ cells were gated and distributed in double plots for IgD and IgM expression. Frequencies of the different B cell populations inside the CD19^+^ population were determined by the differences in the expression of these markers, as follows: Naive B cells (IgMlo/hiIgDhi, Naïve B); Marginal Zone-type B cells (IgMhiIgDlo, MZB-type); Transitional B cells (IgMlo/hiIgD-/lo, Transitional B) and “Switched”/memory B cells (IgM-IgD-,“Switched”/Mem B). The figure shows examples of plots obtained with a normal, uninfected mouse or 20 d.p.i. mouse inoculated with 102 bloodstream forms of *T. vivax*.
(0.37 MB TIF)
