# Supplementary figures and images for: Trypanosoma vivax Infections: Pushing Ahead with Mouse Models for the Study of Nagana. II. Immunobiological Dysfunctions
Source: PLoS Negl Trop Dis. 2010 Aug 10;4(8):e793. doi: 10.1371/journal.pntd.0000793 (PMC2919407; doi:10.1371/journal.pntd.0000793)

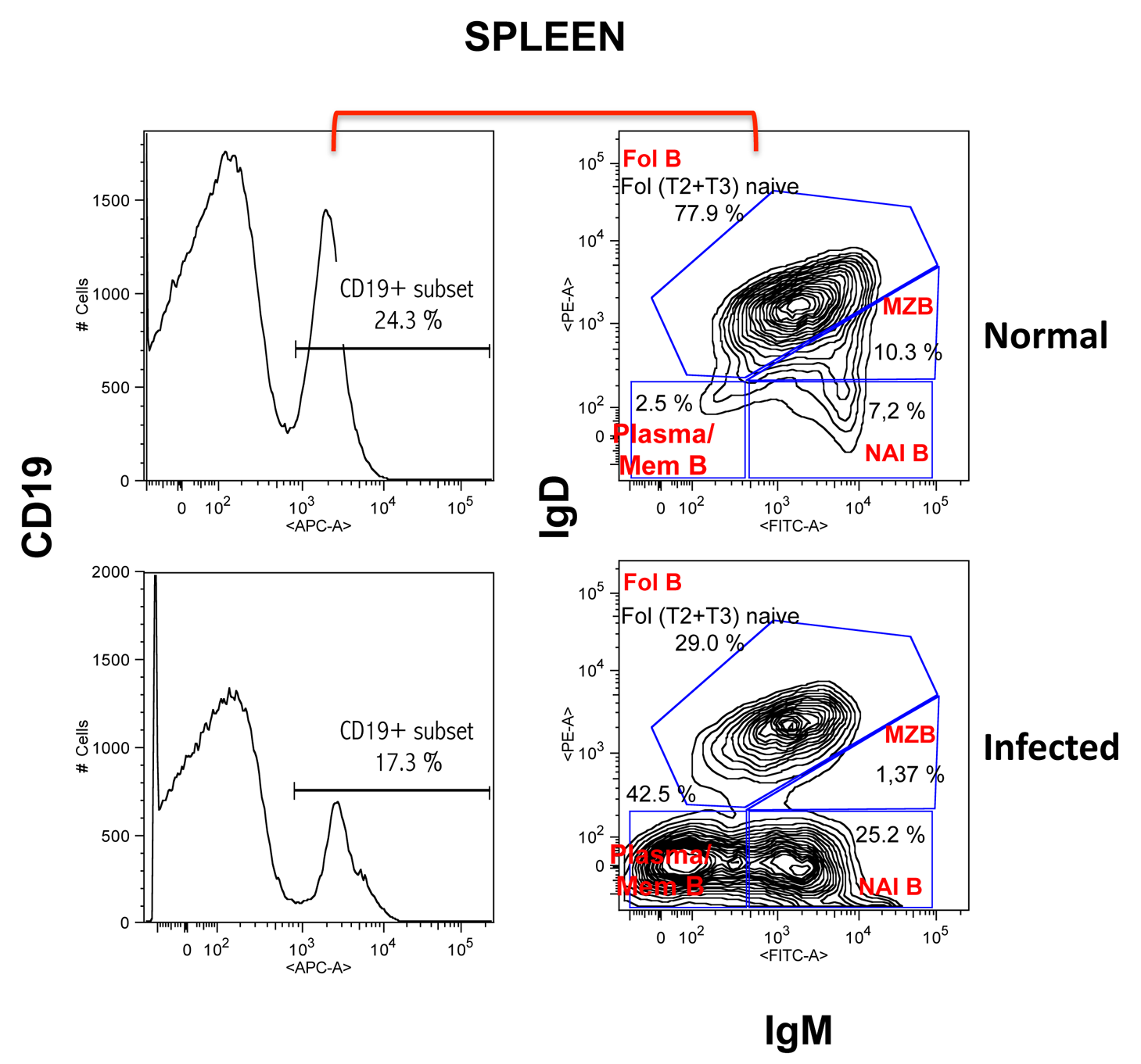

Supplement: Figure S1 — Gating strategy for the analysis of B cell populations in the spleen. Spleen cells were stained with CD19, IgM and IgD antibodies. 105 events were acquired in a FACScanto (BD biosciences). Lymphocytes were analyzed inside a combined FCS/SSC gate. CD19+ cells were gated and distributed in double plots for IgD and IgM expression. Frequencies of the different B cell populations were determined by the differences in the expression of these markers inside the gated population, as follows: Newly arrived immature B cells (IgMhiIgD-/lo, NAI B); Marginal Zone B cells (IgMhiIgDlo, MZB); Follicular B cells (IgMlo/hiIgDhi, Fo B) and Plasma/memory B cells (IgM-/loIgD-, Plasm/Mem B). The figure shows examples of plots obtained with a normal, uninfected mouse or 20 d.p.i. mouse inoculated with 102 bloodstream forms of T. vivax. (0.38 MB TIF) [file pntd.0000793.s001.tif]

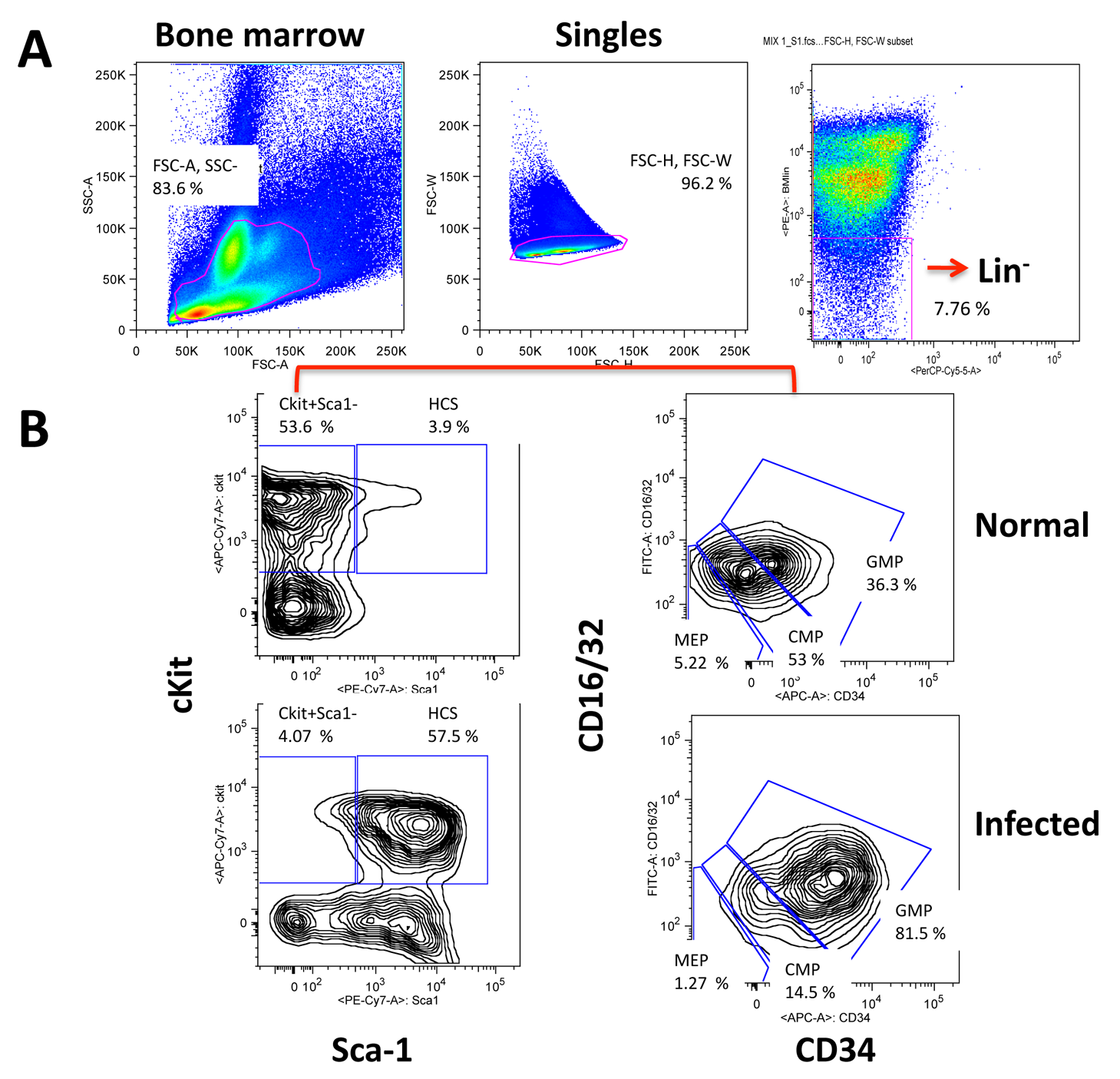

Supplement: Figure S2 — Gating strategy for the analysis of bone marrow cell lineages and progenitors. Bone marrow (BM) cells obtained from 2 femurs were stained with a combination of antibodies for multi-parameter flow cytometry (see Materials and Methods). Briefly, 100000–150000 events were acquired in a FACScanto (BD biosciences). (A) Combined FSC-A/SSC-A and FSC-H/FSC-W gates were used to gate single BM cells. Cells were then analyzed for the expression of lineage markers (lin). Hematopoietic stem cells (HSC) and early progenitors were gated within the lin-/lo fraction as they do not express, or express low levels of mature cell markers. (B) Frequencies of Lin-/lo BM cells were further analyzed for the expression of Sca1 and c-Kit. LSK (lin-/loSca1+cKit+) fraction contains the HSC and was used to determine the frequencies of short and long reconstituting cells by their expression of CD34 (not shown). Identification of progenitor populations was performed using the lin-/loSca1+cKit+ BM gated cells and their expression of CD16/32 and CD34, as follows: Granulocyte/macrophage progenitors (CD16/32hi/CD34hi, GMP); Common myeloid progenitors (CD16/32lo/CD34hi, CMP) and Magakaryocyte/erythrocyte progenitors (CD16/32-CD34-, MEP). The figure shows examples of plots obtained with a normal, uninfected mouse or 20 d.p.i. mouse inoculated with 102 bloodstream forms of T. vivax. (0.69 MB TIF) [file pntd.0000793.s002.tif]

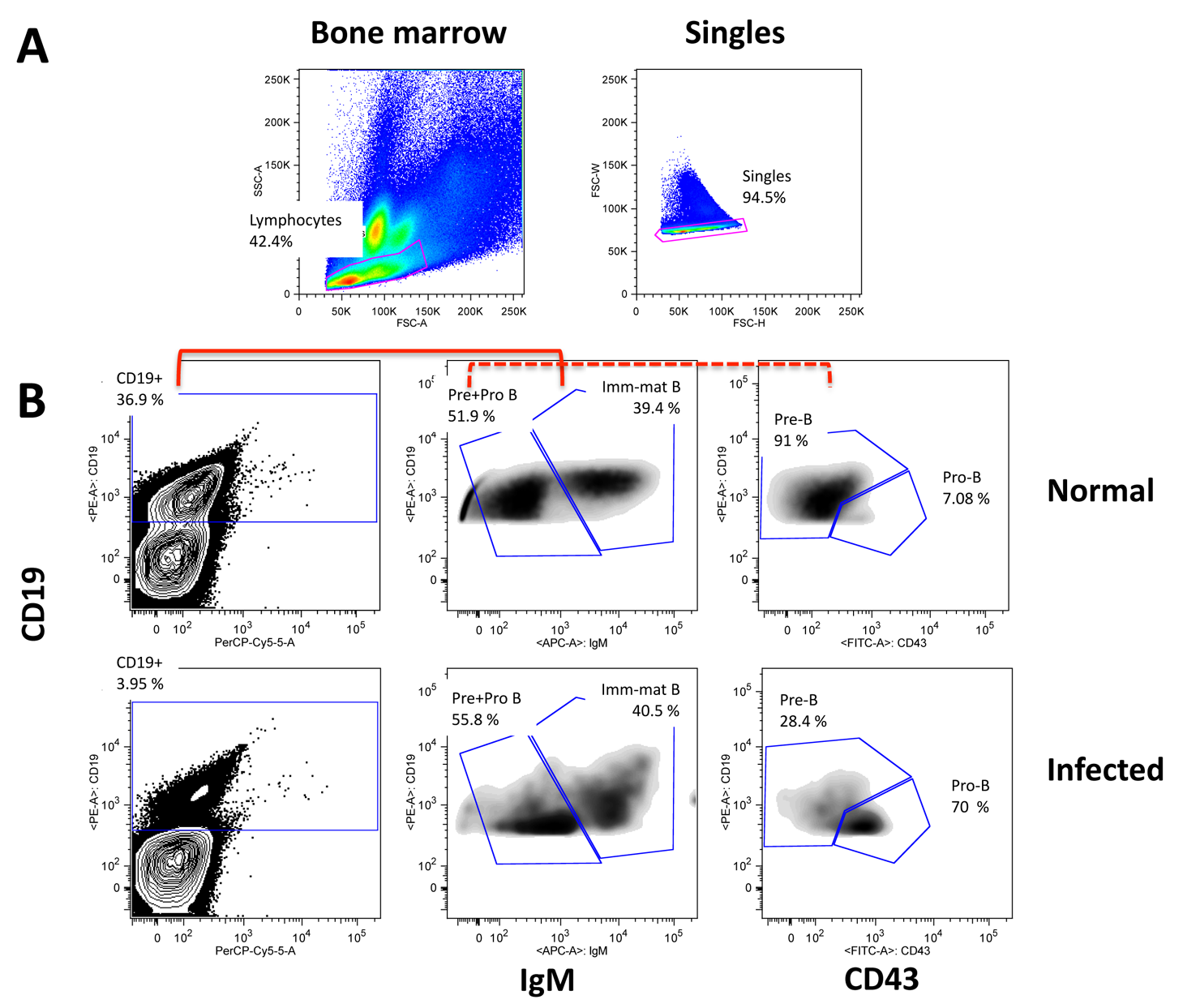

Supplement: Figure S3 — Gating strategy for the analysis of bone marrow cell B cell precursors. Bone marrow (BM) cells obtained from 2 femurs were stained with a combination of antibodies for multi-parameter flow cytometry (see Materials and Methods). Briefly, 100000–150000 events were acquired in a FACScanto (BD biosciences). (A) Combined FSC-A/SSC-A and FSC-H/FSC-W gates were used to restrain the analysis to single BM lymphoid cells. BM cells were further analyzed on the basis of lineage markers (lin). (B) lin+ fraction was then distributed for the expression of CD19. CD19+ cells were gated and frequencies of Pre+Pro B cells and late immature/mature B cell populations were identified on the basis of IgM expression inside the gated population. The expression of CD43 by Pre+Pro B gated cells (lin+CD19+IgM-) gave rise to the determination of Pre-B (CD43−) and Pro-B (CD43+) cell frequencies. The figure shows examples of plots obtained with a normal, uninfected mouse or 20 d.p.i. mouse inoculated with 102 bloodstream forms of T. vivax. (0.48 MB TIF) [file pntd.0000793.s003.tif]

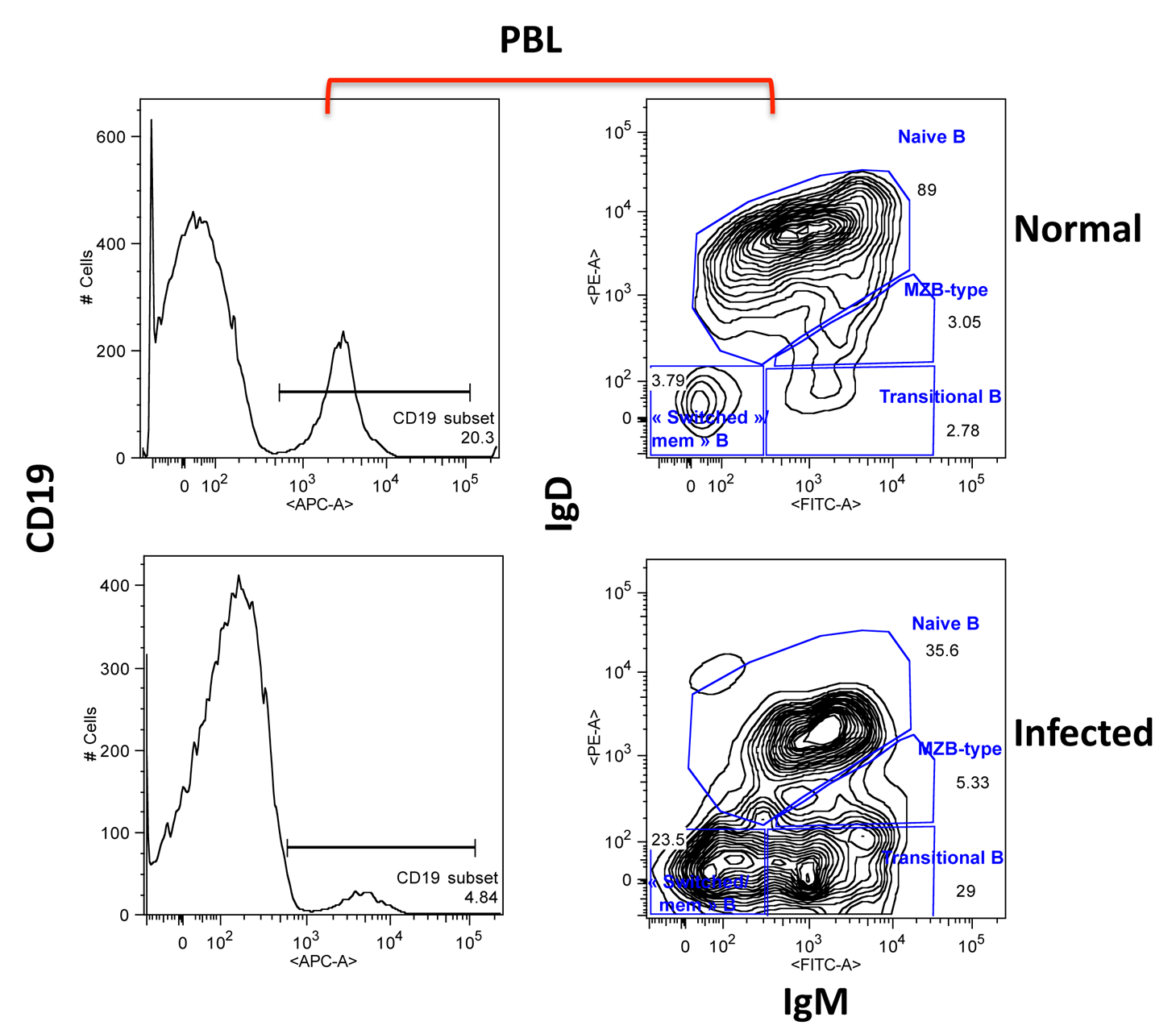

Supplement: Figure S4 — Gating strategy for the analysis of of B cell populations in blood. PBL cells were stained with CD19, IgM and IgD antibodies. 20000 events were acquired in a FACScanto (BD biosciences). Lymphocytes were analyzed inside a combined FCS/SSC gate. CD19+ cells were gated and distributed in double plots for IgD and IgM expression. Frequencies of the different B cell populations inside the CD19+ population were determined by the differences in the expression of these markers, as follows: Naive B cells (IgMlo/hiIgDhi, Naïve B); Marginal Zone-type B cells (IgMhiIgDlo, MZB-type); Transitional B cells (IgMlo/hiIgD-/lo, Transitional B) and “Switched”/memory B cells (IgM-IgD-,“Switched”/Mem B). The figure shows examples of plots obtained with a normal, uninfected mouse or 20 d.p.i. mouse inoculated with 102 bloodstream forms of T. vivax. (0.37 MB TIF) [file pntd.0000793.s004.tif]
